# Prediction of Molecular Targets of Cancer Preventing Flavonoid Compounds Using Computational Methods

**DOI:** 10.1371/journal.pone.0038261

**Published:** 2012-05-31

**Authors:** Hanyong Chen, Ke Yao, Janos Nadas, Ann M. Bode, Margarita Malakhova, Naomi Oi, Haitao Li, Ronald A. Lubet, Zigang Dong

**Affiliations:** 1 The Hormel Institute, University of Minnesota, Austin, Minnesota, United States of America; 2 The National Cancer Institute, Bethesda, Maryland, United States of America; University College Dublin, Ireland

## Abstract

Plant-based polyphenols (i.e., phytochemicals) have been used as treatments for human ailments for centuries. The mechanisms of action of these plant-derived compounds are now a major area of investigation. Thousands of phytochemicals have been isolated, and a large number of them have shown protective activities or effects in different disease models. Using conventional approaches to select the best single or group of best chemicals for studying the effectiveness in treating or preventing disease is extremely challenging. We have developed and used computational-based methodologies that provide efficient and inexpensive tools to gain further understanding of the anticancer and therapeutic effects exerted by phytochemicals. Computational methods involving virtual screening, shape and pharmacophore analysis and molecular docking have been used to select chemicals that target a particular protein or enzyme and to determine potential protein targets for well-characterized as well as for novel phytochemicals.

## Introduction

The consumption of fruits and vegetables has long been believed to decrease the risk of developing various types of human cancers [1]. A major class of compounds within foods that possess these activities are the polyphenols [2]. The phenylpropanoid metabolic pathways of plants generate these polyphenolic compounds from thousands of secondary plant metabolites [3]. Flavonoids are the most common family of polyphenolic compounds with up to 8,000 individual compounds identified [4]. Flavonoids found in vegetables, cereals, legumes, fruits, and beverages such as wine, teas, and coffees can be subdivided into 14 different categories based on chemical structure. These categories include the chalcones, dihydrochalcones, aurones, flavones, flavonols, dihydroflavonols, flavanones, flavanols, flavandiols, anthocyanidins, isoflavonoids, biflavonoids, and proanthocyanidins [5].

The anticarcinogenic potential of a variety of well-characterized flavonoids has been well documented [1]. Isoflavonoids, such as anthraquinones, chalcones, and prenylflavonoids, are all capable of promoting estrogenic activity in mammals [6]. They have also been shown to possess anticancer properties [7]. Genistein, for example, a major member of the isoflavonoid family derived from soybeans [7], specifically inhibits the epidermal growth factor receptor (EGFR) tyrosine kinase activity, which plays an important role in cell proliferation and transformation [8]. New findings continue to be reported related to these compounds. Resveratrol, a polyphenol found in red wine, is known to possess antioxidant activities as well as anticancer activities explained by its inhibition of the cyclooxygenase proteins [9]. Recently, we reported that resveratrol can suppress leukotriene A_4_ hydrolase (LTA_4_H) activity [10], which is over-expressed in lung and colon cancer cells [11].

Flavonoids are promiscuous in that they can suppress the growth of many different types of cancer cells through a variety of mechanisms. This nonspecificity is compounded by the fact that thousands of flavonoids exist and therefore their study has provided a very rich area of research. Only a few cases of computational work focusing on flavonoids exist [12], [13], [14]. Using conventional methods to select the best single or group of best chemicals for identifying compounds that are effective in on treating or preventing a disease like cancer is difficult. Computational strategies for determining protein targets of flavonoids have not yet received a great deal of attention. Over the last 3 years, our laboratory has utilized computational strategies that include virtual screening, shape similarity-screening, and molecular docking to identify potential protein targets of flavonoids and other phytochemicals [15]. These computational-based methodologies have provided efficient and inexpensive tools to gain further understanding of the anticancer and therapeutic effects exerted by polyphenols. Herein we present our process for combining those computational strategies with experimental methodologies for validating specific flavonoids and their respective protein targets.

## Materials and Methods

### Virtual Screening

Virtual screening is a computational technique used in drug discovery research in recent years and it has become an important step in the drug discovery process. The screening involves the identification and compilation of relevant chemical structures from large chemical libraries. The chemicals identified are those most likely to bind to a protein target, typically a protein receptor selected by using various computer programs or identified experimentally. Virtual screening by molecular docking is the major computational method employed in drug discovery for “hit” identification [16]. The primary methodology used is structure-based virtual screening, which involves docking of thousands of candidate ligands into a protein target followed by scoring the protein-ligand binding interaction to estimate the binding energy of the ligand [17]. Structure-based virtual screening requires the 3D structure of the ligands. The ZINC (i.e., an acronym for “ZINC is not commercial”) Database contains over 13 million purchasable compounds in 3D docking format that are freely available for virtual screening [18]. From this huge database, smaller and more specific high-quality libraries can be built for targeted virtual screening [19]. Another available database containing 2D forms of molecules is the National Institute of Health’s PubChem online database comprising over 27 million unique structures (http://pubchem.ncbi.nlm.nih.gov/). In our laboratory, we have created a smaller flavonoid compound database of 2,620 compounds, including aurones, chalcones, flavones, flavanones, isoflavones, biflavonoids, anthocyanidins, dihydrochalcones and proanthocyanidins. These flavonoid compounds were collected and compiled from the NCI PubChem database using structure based searching. This database was used in the screening for potential inhibitors targeting a number of cancer-related proteins, including p90 ribosomal S6 kinase 2 (RSK2), cyclin dependent kinase (Cdk), mitogen-activated protein kinase kinase 1 (MEK1), epidermal growth factor receptor (EGFR) and phosphatidylinositol 3-kinase/protein kinase B (PI3-K/PKB).

**Table 1:** Results of virtual screening for RSK2 inhibitors.

Compound Name	Docking Score (kcal/mol)	Activity Validation
Pedalitin	−10.20	*
Quercetin 3-sulfate	−9.93	*
Quercetin	−9.40	Yes
5-Hydroxy-4′-methoxy-7-methylflavone	−9.28	*
Kaempferol	−8.86	Yes
3,3′-di-O-ethylquercetin	−8.50	*

* , Not commercially available.

**Figure 1:**
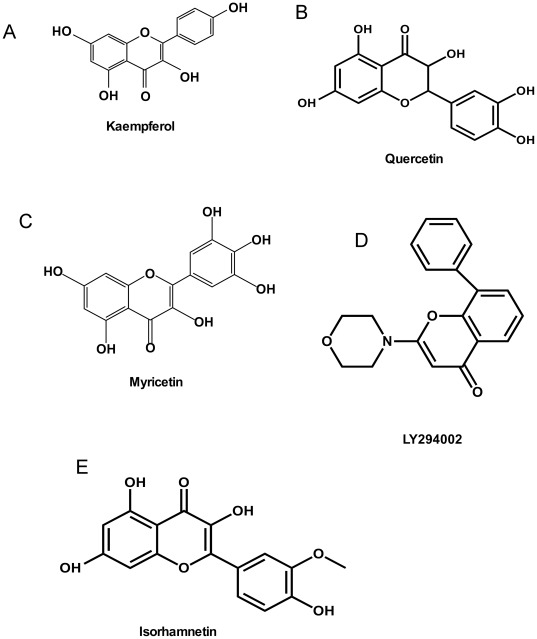
Chemical structures. (A) Chemical structure of kaempferol; (B) Chemical structure of quercetin. (C) Chemical structure of myricetin. (D) Chemical structure of LY294002. (E) Chemical structure of isorhamnetin.

The Ras/extracellular signal-regulated kinase (ERK) pathway regulates cell proliferation, survival, growth and motility and tumorigenesis [20]. RSK2, a member of the p90RSK family, is a direct substrate kinase of ERKs and is an important direct effector for transcriptional activation of downstream target transcription factors. Furthermore, RSK2 is reportedly involved in prostate cancer cell proliferation [21] and *c-fos*–dependent osteosarcoma development [22]. RSK2 protein abundance is increased in many human cancer cell lines and in various human skin tumors, including melanomas and squamous cell carcinomas [23]. Therefore, identifying a selective RSK2 inhibitor is extremely important for chemoprevention or therapeutic drug development. Therefore, herein we used RSK2 as an example to demonstrate how computational strategies and experimental methodologies can be combined effectively to identify selective flavonoid inhibitors. To date, a number of potential RSK2 inhibitors have been reported, including eriodictyol [24] and kaempferol [23], [25], [26], two flavonoid compounds and SL0101, a synthesized compound not found in plants [27]. These flavonoids are ubiquitously found in fruits and vegetables as well as popular beverages, including wine, tea, and coffee and exhibit antioxidant, antitumor, and anti-inflammatory effects [28].

**Figure 2:**
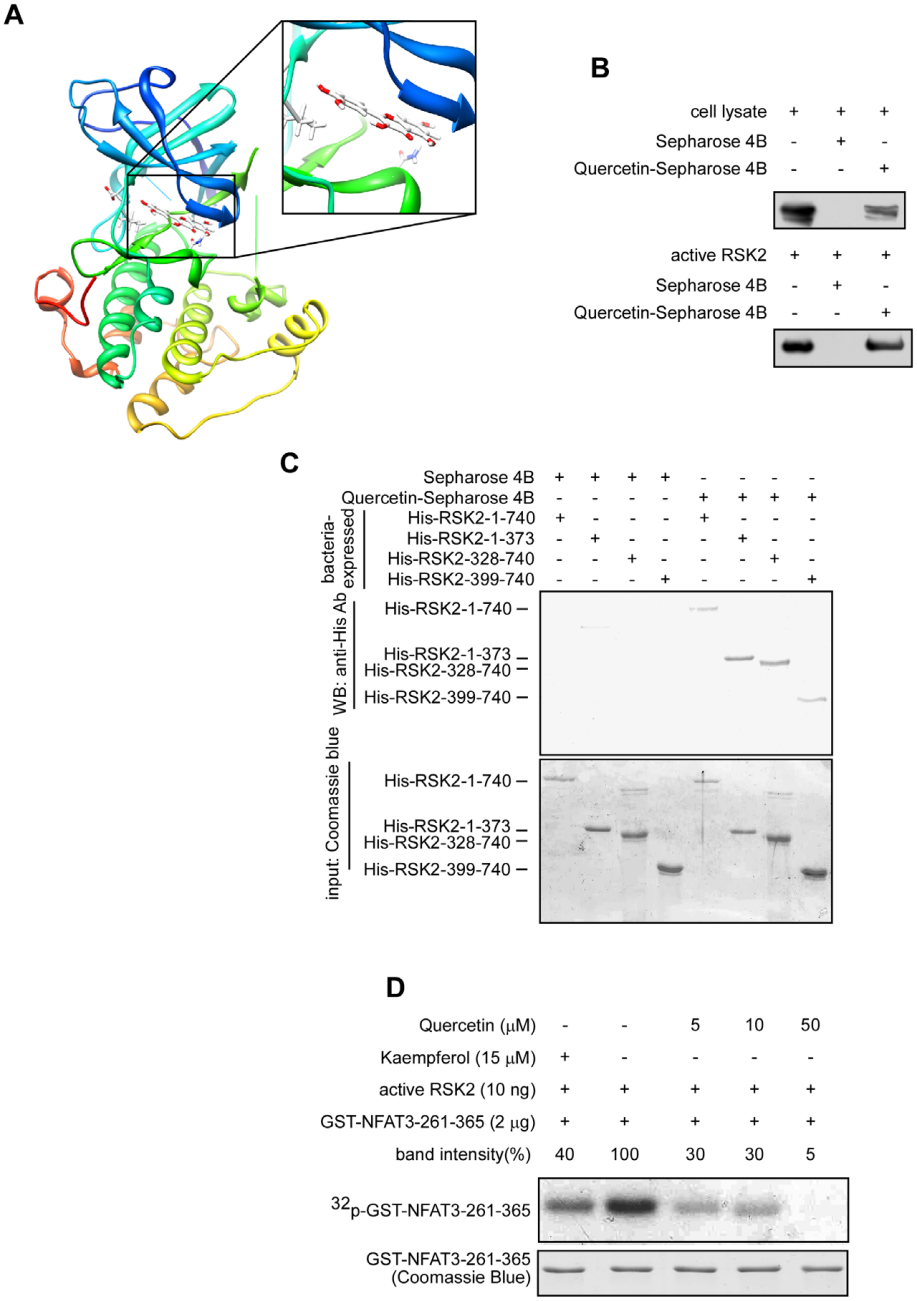
Quercetin binds with RSK2 and inhibits RSK2 activity *in vitro*. (A) Quercetin binds at the ATP pocket of RSK2 most likely in an ATP-competitive manner. (B) RSK2 binds with quercetin. A lysate prepared from JB6 C41 cells or commercially available active RSK2 was incubated with Sepharose 4B-quercetin beads or with Sepharose 4B beads alone, and the pulled down proteins were analyzed by Western blot. (C) Quercetin binds with either the NTD or the CTD of RSK2. To identify the RSK2 domain that binds with quercetin, RSK2 proteins, as indicated, were incubated with Sepharose 4B-quercetin beads or with Sepharose 4B beads alone. The pulled down proteins were analyzed by Western blot. (D) Active RSK2 (10 ng) was combined with GST-NFAT3-261-365 (2 µg), 10 µM unlabeled ATP, 10 µCi [γ-^32^P]ATP, and different doses of quercetin (0–50 µM). An *in vitro* kinase assay was performed and the^32^P-labeled phosphorylated NFAT3 was visualized by autoradiography. Band density was quantified using the Image J software program (NIH) and the band intensity of active RSK2 and GST-NFAT3-261-365 (100%) was compared.

Our laboratory has solved and reported the crystal structures of the C-terminal and N-terminal kinase domains of RSK2 [29], [30]. The N-terminal kinase domain was bound with ANP at the ATP binding site. Thus, this structure (PDB ID:3G51) was downloaded from the PDB Bank for virtual screening studies. Crystal structures or homology models of the target protein to which a small molecule will be docked are downloaded from the Protein Databank (PDB). Waters, metals, and ligands are then stripped from the structure and hydrogens and atom charges are added to the structures using the protein preparation module in Schrödinger’s Maestro v9.2 GUI. An ATP binding site-based pocket was generated within a 30-Å^3^ grid. The 2D structure database of flavonoids was converted to a 3D structure database using the LigPrep module of the Schrödinger Suite of software.

**Table 2:** Potential kinase targets of myricetin.

Protein	PDB ID	Ligand Code	Shape SimilarityScore	Reported Inhibitor	Average SimilarityScore	Hits
Pim-1	2O3P	QUE	0.90	yes	0.82	8**
	2O63	MYC	0.88	yes		
	2O64	MYU	0.86	yes		
GSK-3β	3DU8	553	0.83	yes	0.79	5**
	1Q41	IXM	0.80	yes		
	3ZRL	ZRL	0.78	yes		
PI3-K*	3PRZ	3RZ	0.81	yes	0.77	6**
	3DPD	41A	0.79	yes		
	3PS6	3PS	0.78	yes		
Cdk2	1E1V	CMG	0.83	yes	0.76	16**
	1H0W	207	0.82	yes		
	1E1X	NW1	0.80	yes		
Raf*	3PPJ	FOI	0.73	yes	0.72	2
	3C4C	324	0.71	yes		
MEK1*	3EQH	ADP	0.71	yes	0.71	1

* , Protein targets that been validated in our laboratory.

** , Only the top 3 hits are shown here.

High throughput virtual screening (HTVS) and docking are usually performed first because they are intended for the rapid screening of large numbers of ligands followed by standard and extra precision (SP and XP) docking. Here, for our flavonoid database, only SP and XP docking were performed because of the smaller number of ligands involved. All compounds were docked flexibly and a top-20 list of compounds was generated and organized based on the docking score (i.e., lower score is best). A list of the top-6 ranked compounds was compiled (Table 1) and kaempferol (Fig. 1A) and quercetin (Fig. 1B) were purchased for experimental validation. Kaempferol and quercetin are natural flavonols found in apples, onions and other plants.

**Table 3:** Sixflavonoid candidate inhibitors for PI3-K.

Compound Name	Shape Similarity Score	Validation
Mitoflaxone	0.84	*
Dimeflin	0.83	*
Isowogonin	0.76	*
Kumatakenin	0.76	*
Myricetin	0.75	Yes
Isorhamnetin	0.75	Yes

* , Not commercially available.

### Shape-Similarity Screening Method

The theory of shape-similarity screening is derived from the idea that molecules possessing similar shapes and electrostatic capabilities might exhibit analogous biological activity. The method involves consideration of the atomistic and spatial characteristics of the target molecule. The pharmacophore and physical features of the molecule are quantitatively compared with a library of compounds. When searching for potential target proteins, the compound library used is composed of crystallized ligands extracted from the most recent version of the PDB [31]. The ligand conformation in the crystal structure is used because the atoms are oriented in a manner optimized for binding to the protein. Any available database can be used when searching for similar compounds.

**Figure 3:**
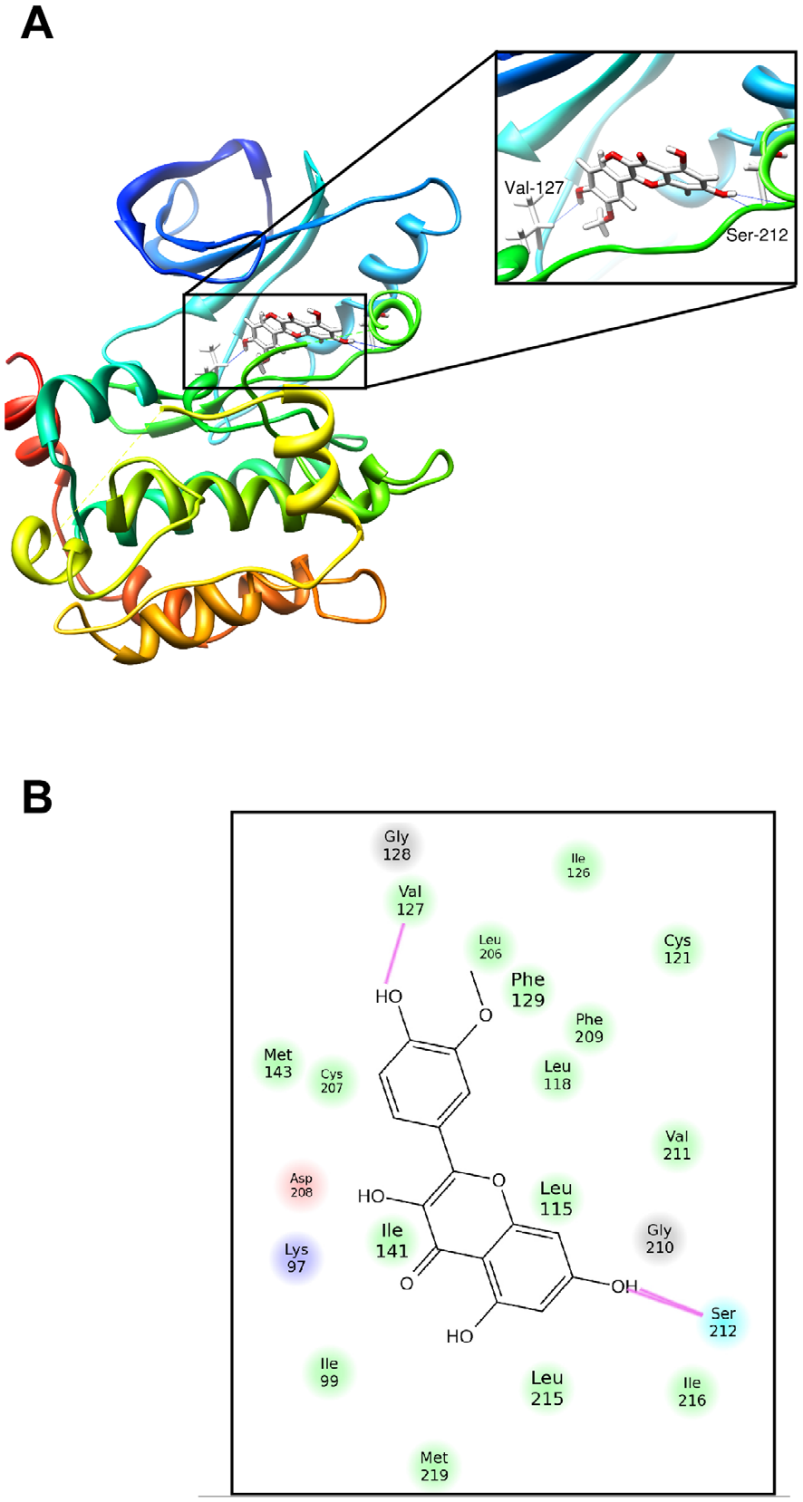
Modeling of isorhamnetin binding with MEK1. (A) Isorhamnetin binds to an ATP-noncompetitive pocket of MEK1. The box indicates an enlarged view. Hydrogen bonds are formed between isorhamnetin and the backbone of MEK1 (Val127 in the ATP-noncompetitive binding site and Ser212 in the activation loop). (B) Ligand interaction diagram of the MEK1 and isorhamnetin complex. Residues are represented as colored spheres, labeled with the residue’s name and number. The colors indicate the residue type (green  =  hydrophobic; blue  =  polar). The solid pink line shows the hydrogen bond between the ligand and the receptor. Hydrophobic interactions are formed with the side chain at Ile99, Phe129, Ile141, Phe209 and Leu118.

The PHASE [32] module of Schrödinger’s molecular modeling software package is one program that can perform this type of shape-similarity search [33]. The atom type information is included for consideration of not only shape similarity but also to align potential pharmacophore points between the queries and targets. The cutoff to be used for the results takes the top aligned structure for each molecule returned and all conformers possessing a Tanimoto similarity coefficient below 0.7 are deleted [34], [35]. The smaller flavonoid compound database that we created and the latest version of the PDB, which contains more than 500,000 protein structures complexed with ligands, were used for shape-similarity screening. We also created a specific kinase database of about 4,000 structures complexed with ligands that we collected from the PDB for screening kinase targets separately.

Our previous studies showed that myricetin (Fig. 1C), a flavonoid found in many grapes, berries, fruits, vegetables, herbs, as well as other plants, and one of the phenolic compounds present in red wine, exhibits potent anticancer and chemopreventive effects, especially on UVB-induced skin cancer [36], but its molecular mechanisms and targets are unclear. Thus, to identify off-target protein affected by myricetin, shape-similarity screening was performed using the PDB ligand database and our special kinase database.

We also attempted to identify novel inhibitors targeting PI3-K, which is a well known kinase involved in cellular functions such as growth, proliferation, differentiation, motility, survival and intracellular trafficking, all of which are associated with cancer development. Wortmannin and LY294002 are broad inhibitors that target all PI3-K family members. LY294002 (Fig. 1D) is a morpholine derivative of quercetin and although its IC_50_ is about 500-fold higher than that of wortmannin, it is still widely used in cell biology as a specific PI3-K inhibitor because of its stability in solution [37]. We therefore used the structure of LY294002 to carry out the screening for potential PI3-K inhibitors in our flavonoid database using the PHASE module of the Schrödinger Suite 2011. To perform the screening, myricetin and LY294002 were first prepared separately using the LigPrep module of the Schrödinger Suite 2011 under the OPLS_2005 force field and a specifying pH value of 7.0. Then shape-similarity screening was carried out using PHASE.

### Molecular Docking Method

Molecular docking has become a standard tool in computational biology for predicting the binding orientation of small molecule drug candidates with their protein targets in order to predict the affinity and activity of the small molecule. Thus, molecular docking plays an important role in the rational design of drugs. GLIDE from the Schrödinger Suite 2011 [38] is one of the programs used in our laboratory to perform docking.

Different protocols for docking are attempted before determining the correct set of parameters to be used for docking. The correct re-docking of the ligand that was crystallized with the target protein is typically used as validation of the chosen parameters. When more than one crystal structure of a target protein is available, cross-docking is performed to determine which crystal structure is most suitable for docking [39].

In our laboratory, we had found that isorhamnetin (Fig. 1E), an O-methylated flavonol in herbal medicinal plants such as red turnip, goldenrod, mustard leaf and gross *Hippophaer hamnoides L.,* could inhibit the kinase activity of MEK1 or PI3-K and the inhibition was due to isorhamnetin’s direct binding with these kinases [40]. Molecular docking was used to further study the binding of isorhamnetin with MEK1. First an X-ray structure of the human MEK1 in a complex with ligand and MgATP (PDB 1S9J) with a 2.4 Å resolution was downloaded from the PDB. The protein was prepared for docking using the Protein Preparation Wizard. All crystallographic waters were deleted and a 30-Å^3^ grid was generated based on the ATP noncompetitive ligand (BBM) binding site of the protein receptor. MacroModel was used to build and energetically minimize isorhamnetin to create the most energetically favorable conformation needed for docking studies.

Several standard procedures included in Schrödinger’s GLIDE docking protocols were performed. Procedures included docking with standard precision (SP) or extra precision (XP) in GLIDE, and the more CPU-intensive Induced-Fit Docking (IFD) method with the default parameters were conducted with SP and XP docking. All these docking procedures allowed ligand docking flexibility and a total of 20 top ranked structures were analyzed using the IFD method.

## Results

### Virtual Screening

Based on our virtual screening results for RSK2, quercetin and kaempferol have binding scores of −9.40 kcal/mol and −8.86 kcal/mol, respectively, with RSK2 (Table 1). These scores are a very good indication of binding compared with −7.73 kcal/mol for SL0101, a well-known RSK2 inhibitor. Our docking model shows quercetin binding in an ATP-competitive manner within the ATP banding site of RSK2 (Fig. 2A), indicating that quercetin might also be a potential inhibitor of RSK2. To examine our hypothesis, we conjugated quercetin with Sepharose 4B beads and conducted an *in vitro* pull-down assay using a whole cell lysate or an active RSK2 protein (200 ng; Fig. 2B). The results showed that the RSK2 protein bound with Sepharose 4B-quercetin beads but not with Sepharose 4B beads alone (Fig. 2B). Furthermore, we conducted a pull-down assay with Sepharose 4B-quercetin beads and several bacterially-expressed His-tagged RSK2 protein fragments, including His-RSK2-1-740, His-RSK2-1-373, His-RSK2-328-740, and His-RSK2-399-740. Western blot results indicated that both the NTD and CTD of RSK2 bound with Sepharose 4B-quercetin beads (Fig. 2C). To confirm the results of the virtual screening that identified quercetin as a potential RSK2 inhibitor, we conducted an *in vitro* kinase assay. Results indicated that quercetin inhibited RSK2 activity in a dose-dependent manner (Fig. 2D). We previously used an *in vitro* kinase assay, an anchorage-independent cell transformation assay and a pull-down assay with Sepharose 4B-kaempferol beads to show that kaempferol binds with the NTD of RSK2 and inhibits RSK2 activity *in vitro* and *ex vivo* [23], [26], [31].

#### Shape-Similarity Screening

In our study, the shape-similarity screening was performed using Schrödinger’s PHASE module to examine the database comprised of protein complexes with ligands and the specific kinase database. Myricetin, a flavonoid found in grapes, berries, fruits, vegetables, herbs, as well as other plants, and a phenolic compounds present in red wine, was determined to target many potential kinases (Table 2). The data indicate six different kinase/ligand complexes with an average score of shape-similarity with myricetin greater than 0.7. The ligand with the greatest similarity to myricetin is QUE or quercetin (Fig. 1B), which is bound with Pim-1 (PDB ID: 2O3P). The similarity score is 0.90 and quercetin is a reported inhibitor of Pim-1 [41]. A total of 8 Pim-1 structures were “hit” by myricetin with a similarity score of greater than 0.7 (only the top 3 hits are shown in Table 2). Almost all the “hit” ligands are reported inhibitors of their targeted kinase and have a similarity score of greater than 0.7. Thus, we believe that these related kinases might be possible targets of myricetin and that myricetin might potentially inhibit their activity. Our previous studies indicated that myricetin could inhibit the activity of PI3-K, MEK1 and Raf [36], [42], [43].

To find potential flavonoid inhibitors of PI3-K, LY294002, a broad PI3-K inhibitor, was chosen to use as the query structure and shape-similarity screening was performed using our flavonoid database and the PHASE module. Similarity coefficients below 0.75 were deleted and 6 of the top ranked candidate compounds are listed (Table 3). We have previously validated myricetin [42] and isorhamnetin [40] as direct inhibitors of PI3-K.

### Molecular Docking

To examine the molecular mechanism of the inhibition of MEK1 by isorhamnetin and to understand how isorhamnetin interacts with MEK1, we docked isorhamnetin *in silico* to the ATP-noncompetitive binding pocket of MEK1 using several protocols in the Schrödinger Suite of software. By studying all the models returned, we found that isorhamnetin formed some favorable connections and docked nicely within the MEK1 ATP-noncompetitive binding site. Some important hydrogen bonds were formed between isorhamnetin and the backbone of MEK1, including Val127 in the ATP-noncompetitive binding site and Ser212 in the activation loop. Isorhamnetin also formed hydrophobic interactions with the side chain at Ile99, Phe129, Ile141, Phe209 and Leu118 (Fig. 3A,B). These results would render MEK1 catalytically inactive by stabilizing the inactive conformation of the activation loop. Note that some images were generated with the UCSF Chimera program [44].

## Discussion

Thousands of individual flavonoid compounds exist in various vegetables, fruits, and other plants. Flavonoids, such as catechins found in strawberries and green and black teas, kaempferol from brussel sprouts and apples, and quercetin from beans, onions and apples, are believed to exert anti-inflammatory and anticancer activities. For example, all of these compounds reportedly might reduce the risk of developing lung cancer [45]. Flavonoids inhibit many different types of cancers through a variety of mechanisms and thousands of flavonoids exist, which makes the screening of their potential anticancer effects and identification of their specific protein targets extremely challenging using conventional approaches. Fortunately, the development of computational simulation techniques and other computational strategies has simplified and streamlined the overall process. Virtual screening can easily generate results from all flavonoid compounds that can bind with and affect the activity of a specific protein target. Shape-screening can assist in finding potential “off-target” proteins of a specific flavonoid compound. Molecular docking methods can provide a better indication of how a compound interacts with its protein target and influence the activity of the targeted protein. These three processes can lead to the rapid discovery of potential lead compounds for anticancer treatment and chemoprevention.

In recent years, with the help of the Blue Gene/L [46], [47] supercomputer from IBM, our laboratory has studied the effectiveness and mechanisms of several lead flavonoid compounds in cancer chemoprevention and treatment by using computational simulation strategies. These compounds include kaempferol [23], isorhamnetin [40], 6,7,4′-trihydroxyisoflavone [48], eriodictyol [24], 7,3′,4′-trihydroxyisoflavone [49], quercetin [50], EGCG [51], [6]-gingerol [52], myricetin [36], coffee phenolic phytochemicals [53], caffeic acid [54] and delphinidin [55]. All these compounds exert their inhibitory effect on specific proteins, including RSK2, PI-3K, MEK1, Pin-1 and Fyn, which are highly expressed or overactivated in some cancers such as skin and colon cancer.

To identify the potential targets of a variety of flavonoid compounds and to carefully study their mechanism of action, we created a flavonoid database of 2,620 compounds. We are continuing to enlarge the database by adding more and more flavonoid compounds. Based on this database, we have obtained results for every compound and its potential off-target proteins by using shape-similarity screening. We have more than 4.1 million records for all kinds of protein targets and 374,000 records for the specific kinase targets. These results can be easily queried by compound identification or by protein type. We have also screened for lead compounds targeting several specific kinase targets related to skin cancer, colon cancer and lung cancer using virtual screening and will use these results for further mechanistic studies in cancer chemoprevention and therapy.
